# Temperature clusters in commercial buildings using k-means and time series clustering

**DOI:** 10.1186/s42162-022-00186-8

**Published:** 2022-02-22

**Authors:** Ashani Wickramasinghe, Saman Muthukumarana, Dan Loewen, Matt Schaubroeck

**Affiliations:** 1 Department of Statistics, Faculty of Science, University of Manitoba, Winnipeg, MB R3T 2N2 Canada; 2 ioAirFlow, 3rd Floor, 303 Portage Avenue, Winnipeg, MB R3B 2B4 Canada

**Keywords:** K-means clustering, Time series clustering, Machine learning, Building science, Thermostats, HVAC zoning optimization

## Abstract

An efficient building should be able to control its internal temperature in a manner that considers both the building’s energy efficiency and the comfort level of its occupants. Thermostats help to control the temperature within a building by providing real-time data on the temperature inside that space to determine whether it is within the acceptable range of that building’s control system, and proper thermostat placement helps to better control a building’s temperature. More thermostats can provide better control of a building, as well as a better understanding of the building’s temperature distribution. In order to determine the minimum number of thermostats required to accurately measure and control the internal temperature distribution of a building, it is necessary to find the locations that show similar environmental conditions. In this paper, we analyzed high resolution temperature measurements from a commercial building using wireless sensors to assess the performance and health of the building’s HVAC zoning and controls system. Then we conducted two cluster analyses to evaluate the efficiency of the existing zoning structure and to find the optimal number of clusters. K-means and time series clustering were used to identify the temperature clusters per building floor. Based on statistical assessments, we observed that time series clustering showed better results than k-means clustering.

## Introduction

In commercial buildings, it is hard to maintain and control the environment of the building while considering thermal comfort and energy consumption. These buildings are usually equipped with intelligent HVAC (heating, ventilation, and air conditioning) systems. Thermostats send data about the temperature of a space (or zone) within a building to the HVAC system, which then adjusts the air temperature supplied to that space accordingly. Fewer thermostats installed generate a less complete picture of a building’s performance, as different spaces (zones) can have different temperature needs. Thermostats that are improperly placed can also yield incomplete measurements, causing HVAC systems to run too often or not frequently enough. As a result, the temperature measured by the thermostats might be very different from what the building’s occupants are experiencing.

The consequences of inefficient HVAC systems can have serious effects on a building and its occupants. People can have adverse impacts on their productivity and cognitive abilities. For example, a direct correlation has been determined that each 4 degrees Fahrenheit shift away from the optimal internal temperature of 72 degrees resulted in a $2\%$ decrease in productivity. Notably, the economic impact of this productivity decrease also demonstrated that regaining that $2\%$ productivity increase yields a $9\%$ increase in net revenue for a company working within that environment.  (Allen and Macomber 2020) Another study conducted in New York City identified that schools with an internal temperature of 90 degrees Fahrenheit saw a $14\%$ higher likelihood of failing an exam than if that same space was controlled to 75 degrees  (Allen and Macomber 2020).

Nowadays, much research has been done on the topic of building health and thermal comfort. In 2017  (Lee et al. 2017) a research was done to identify the thermal comfort of a residential house in Malaysia and found that to satisfy human thermal comfort HVAC system is needed for the bedroom and living room. Martin Sarnsvosky and David Bajus  (2017) used k-means clustering to cluster the university building based on temperature and humidity data which were obtained by sensors. With the development of smart technologies, people tend to build smart thermostats to control HVAC systems. Smart thermostats can collect more information than traditional thermostats and use machine learning algorithms to optimize the setpoint based on both efficiency and occupant comfort. Hence in 2012 research was done to build a smart energy system to control HVAC systems based on temperature and humidity  (Yun and Won 2012). This system was evaluated using occupants' feedback. But not all building owners are concerned with replacing their existing thermostats with smart devices, due to their high capital cost and how difficult they can be to properly install. A preferred solution would be to find ways to optimize a building’s controls system using its existing infrastructure, allowing thermostats to be deployed and used more efficiently. Hence, our research work is focused on giving a solution to that.

Nikolaou and team  (2012) have used five clustering techniques: Hierarchical, K-Means, Gaussian Mixture Models, Fuzzy, and Neural algorithms to cluster energy and thermal comfort of office buildings. They have not considered time series clustering though. Another research work  (Adán et al. 2020) presents a temporal-clustering based technique to identify thermal regions of buildings using a set of thermal orthoimages (STO). To test this technique in real life they have used 3D thermal scanners. Time series based clustering on temperature using Gaussian Mixture Model was used here, (Westermann et al. 2019) but it was done using only one variable.

In our study, we have clustered each building floor based on temperature data which were collected from wireless sensors using k-means and time-series clustering. In the k-means method, we clustered sensors based on mean values of variables, while in the time series clustering method, we clustered the sensors that show similar trends over time. We also considered temperature, humidity, and pressure variables for clustering and compared the cluster results with each other. Placing a minimum of one thermostat in each identified cluster will generate more accurate measurements and may lead to better control of a building’s thermal performance.

## Material and methods

### Data collection and preparation

The data collection was done in a commercial building in downtown Winnipeg, MB, Canada. We selected three different floors (first, second and fourth floors) which were considered as problematic floors by the building owner. Sixty (60) sensors were strategically positioned throughout these floors and air data (temperature, relative humidity, and pressure) was collected at five minute intervals between February 1 and February 9, 2021. During that period, over 135,000 data points were collected to evaluate the building’s performance. Weather data was collected using the Government of Canada’s weather API. A sample of sensor data set and weather data set is shown in Tables 1 and 2 respectively.

**Table 1:** Sample of sensor data set

Time	Sensor number	Temperature (°C)	Humidity (%)	Pressure (kPa)
2021-02-09 08:58:43	1	26.4	6	99.19
2021-02-09 08:58:42	2	20.3	6	99.15
2021-02-09 08:58:37	3	20.4	6	99.16
2021-02-09 08:58:34	4	20.7	6	99.18

**Table 2:** Sample of weather data set

Time	Temperature (°C)	Relative humidity (%)	Pressure (kPa)
2021-02-09 08:00	− 24.6	61.0	99.20
2021-02-09 09:00	− 23.8	58.0	99.22
2021-02-09 10:00	− 23.3	52.0	99.28
2021-02-09 11:00	− 22.9	52.0	99.31

Based on the data, we could understand that not all 60 sensors started to collect data at the same time. For example, when the first sensor started to collect data at “08:00:45”, the second one started at “08:00:51”. Hence there was a few second differences between the data points of each sensor. It created null data points when we considered all time points as not all the sensors have data at each time point. To fix this, we rounded seconds into the closest minute, and in the timestamp, we considered only the hours and minutes. Also, another issue was that the weather data were collected on an hourly basis. Because of that, when merging weather data with sensor data we were losing most of the valuable data points. We interpolated the weather data to one minute intervals which helped merge the two data sets without losing any data in the sensor data set.

### K-means clustering

In this analysis, we filtered sensors on each floor and considered the mean temperature of each sensor to cluster the sensors. The main objective of clustering is to group similar data points (mean temperature of each sensor) together and discover the underlying pattern. To achieve this goal, k-means requires a fixed number of clusters (k). This target number k, is referring to the number of centroids, which is an imaginary or real location of the center of the cluster. Then every data point is allocated to the nearest cluster while minimizing the intracluster variation.

In the standard k-means clustering algorithm  (Hartigan and Wong 1979) total within-cluster variation is defined as the sum of squared distances of Euclidean distances between items and the corresponding centroid which is shown as:

1 $$\begin{aligned} W(C_k) = \sum _{x_i\epsilon C_k}(x_{i}-\mu _k)^2 \end{aligned}$$

Here $x_i$ is $i^{th}$ data point of cluster k ($C_k$), and $\mu _k$ is the mean value of points in cluster k. The total within-cluster variation is defined as Eq. (2). The total within-cluster sum of squares measures the goodness of the clustering, which increases as the sum of squares measures decreases.

2 $$\begin{aligned} total \;within\;cluster\; variation = \sum _{k=1}^{k}W(C_k)=\sum _{k=1}^{k}\sum _{x_i\epsilon C_k}(x_{i}-\mu _k)^2 \end{aligned}$$

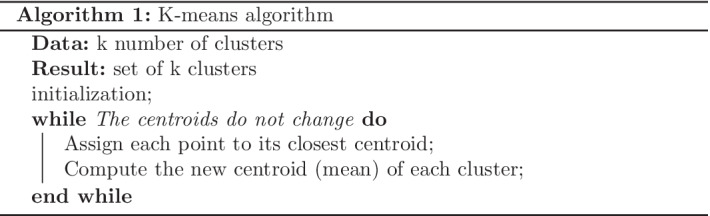


The above algorithm 1 shows the process of K-means clustering; first, specify the number of clusters (k) and second, randomly select k data points as initial centroids. Then assign the remaining data points to their closest centroid. The fourth step is recomputing the new centroids and repeating the third and fourth steps until no changes in centroids.

**Fig. 1:**
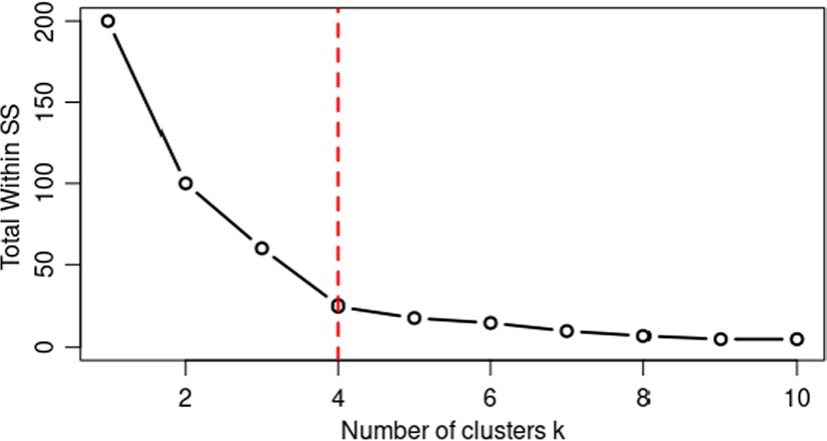
Example for Elbow plot to check optimal number of clusters (k). Red line indicates the elbow joint and it will help to find k value

In this analysis, our objective is to find the optimal number of thermostats for one floor and to achieve that, we needed to find the optimal number of clusters. For that, there are different methods, and we used the elbow plot method. Here we conducted clustering using different numbers of clusters (k) and calculated the total within the sum of squares for each k value and plot it against k. Finally, the k value at the location of bend (elbow joint) in the plot is considered the optimal number of clusters. For an example, Fig. 1 represents an elbow plot, and the total sum of squares distance decreases as k increases, but at k = 4 there is a bend. It shows that having additional clusters will reduce the sum of squares by small values. Hence four can be considered as the optimal number of clusters.

### Time series clustering

In k-means clustering, we considered the mean temperature of each sensor as data points, but by averaging data, potentially valuable information is lost. In time series clustering we can overcome that issue by considering all data points and grouping sensors with similar time series into the same cluster. Here, hierarchical clustering is used to cluster the time series based on euclidean distance.

Hierarchical clustering produces a nested hierarchy of similar groups of objects, according to a pairwise distance matrix of the objects  (Nielsen 2016). In time series clustering, objects are series of numbers. In the agglomerative algorithm, clusters are initialized with each series that belongs to their own groups. The algorithm then merges the similar groups into larger clusters, based on the distance matrix. There are several types of methods and distance matrices to develop clusters. Hierarchical clustering does not require the number of clusters to generate clustering results.

The clustering method used by Santos and team  (2019), showed that the mean linkage method using the correlation similarity metric provides the most appropriate results when studying weather variables. This method overcomes some deficiencies of other hierarchical methods in clustering homogeneous groups. Therefore, clustering is less affected by a typical observation in cluster formation, according to Unal et al.  (2003). The average distance between all pairs of objects in any two clusters can be calculated as follows:

3 $$\begin{aligned} D(r,s)=\frac{1}{n_{r}n_{s}}\sum _{i=1}^{n_r}\sum _{j=1}^{n_s}D(x_{ri} - x_{sj}) \end{aligned}$$

where *D*(*r*, *s*) is the distance between clusters r and s, and $(n_r,n_s)$ are the number of elements in those cluster. The main objective of that study was to cluster precipitation data based on their behavior over time by incorporating their temporal variations  (Unal et al. 2003).

In our study, we used the average method and correlation similarity metric based on the work by Unal et al  (2003). To compare those results, we used Ward’s method and Euclidean distance, which is the most popular combination of method and similarity metric for hierarchical clustering. Ward’s method creates groups while minimizing the pooled within-cluster sum of squares. The Euclidean distance between two different time series is called *Q*, and *C* can be calculated as Eq. (3). Here $q_i$ and $c_i$ represent $i^{th}$ data points of time series Q and C respectively:

4 $$\begin{aligned} D(Q,C)=\sqrt{\sum _{i=1}^{n}(q_i - c_i)^2} \end{aligned}$$

#### Multivariate clustering

In multivariate clustering, we try to cluster sensors, where all the features within each cluster are as similar as possible. In this study, we measured temperature, relative humidity, and pressure. Finding an appropriate method to combine those variables for the time series clustering was one of the challenges in this study.

Since time series clustering uses time series to group objects, we needed to create a time series by considering multiple features. For that we proposed the following method to create a single time series by considering all variables. In this process, we first normalized all the series (temperature, relative humidity,and pressure) using Min-Max normalization:

5 $$\begin{aligned} V_{norm}(i)= & {} \frac{v_{i}-Min(v)}{Max(v) - Min(v)} \end{aligned}$$

6 $$\begin{aligned} new_{(i)}= & {} \frac{\sum _{j=1}^{n}(V_{norm}(ji))}{n} \end{aligned}$$

By normalizing the data we brought all the variables into one scale. Then, using Eq. 6, we generated a new variable by combining all three variables. Let $V_{norm}(ji) = i^{th}$ observation of $j^{th}$ variable, $n =$ number of variables, and $new_{(i)}= i^{th}$ observation of new variable. This new variable generated a time series for each sensor, and we used those time series for the clustering.

### Similarity score measures

Once identified the clusters based on different algorithms, a comparison study can be done using similarity measures. Those indices measure the similarity between cluster results with true labels.

#### Adjusted rand index (ARI)

The Rand Index computes a similarity measure between two clustering by considering all pairs of samples and counting pairs that are assigned in the same or different clusters in the predicted and true clustering. If the number of data vectors for clustering is n, then there are $_nC_2$ pairs. For every example pair, there are three possibilities in terms of grouping. The first possibility is that the paired examples are always placed in the same group as a result of clustering (a). The second possibility is that the paired examples are never grouped together (b). The third possibility is that the paired examples are sometimes grouped and sometimes not grouped together. The RI of two groupings is then calculated by the following formula:

7 $$\begin{aligned} RI = \frac{Count\; of\; Pairs\; in\; Agreement}{Total\; Number\; of\; Pairs}=\frac{a+b}{_nC_2} \end{aligned}$$

RI had one drawback; it yields a high value for pairs of random partitions of a given set of examples. To overcome this drawback, the Rand Index score is then “adjusted for chance” into the Adjusted Rand Index  (Hubert and Arabie 1985) score using the following scheme:

8 $$\begin{aligned} ARI = \frac{RI - Expected\; RI}{max(RI)- Expected\; RI} \end{aligned}$$

The adjusted Rand index is thus ensured to have a value close to 0 for random labeling independently of the number of clusters and samples and exactly 1 when the clusterings are identical (up to a permutation).

#### Normalized mutual information (NMI)

Mutual information (MI) is a measure of the similarity between two labels of the same data. Where $|U_i|$ is the number of the samples in cluster $U_i$ and $|V_j|$ is the number of the samples in cluster $V_j$, the Mutual Information between clustering *U* and *V* is given as:

9 $$\begin{aligned} MI(U,V)=\sum _{i=1}^{|U|}\sum _{j=1}^{|V|}\frac{|U_{i}\cap V_{j}|}{N}log\frac{N|U_{i}\cap V_{j}|}{|U_{i}||V_{j}|} \end{aligned}$$

This metric is independent of the absolute values of the labels: a permutation of the class or cluster label values won’t change the score value in any way. This metric is furthermore symmetric and can be useful to measure the agreement of two independent label assignments strategies on the same data set when the real ground truth is not known. Normalized Mutual Information (NMI)  (Williams et al. 2007) is a normalization of the Mutual Information (MI) score to scale the results between 0 (no mutual information) and 1 (perfect correlation).

#### Adjusted mutual information (AMI)

The baseline value of mutual information between two random clusterings tends to be larger when the two partitions have a larger number of clusters (with a fixed number of nodes). Hence adjusted mutual information (AMI)  (Vinh et al. 2010) will be able to adjust the mutual information (MI) score to account for chance. The AMI between clustering *U* and *V* is given as:

10 $$\begin{aligned} AMI(U,V)=\frac{MI(U,V)-E\left\{ MI(U,V)\right\} }{max \left\{ H(U),H(V)\right\} - E\left\{ MI(U,V)\right\} } \end{aligned}$$

This metric is independent of the absolute values of the labels: a permutation of the class or cluster label values won’t change the score value in any way. Here H(U) and H(V) indicate the entropy associated with the partitioning U and V. The AMI takes a value of 1 when the two partitions are identical and 0 when the MI between two partitions equals the value expected due to chance alone.

### Evaluate cluster results

Once perform the cluster analysis we have to evaluate the cluster result. In this study, we used silhouette score to find the goodness of clustering techniques.

#### Silhouette score

Silhouette score is used to observe the separation distance between the resulting clusters  (Rousseeuw 1987). This measures how close each point in one cluster is to points in the neighboring clusters. To calculate the Silhouette score for each observation, the following distances need to be calculated:

1. Mean distance from the observation to all other observations in the same cluster. Let’s denote it by $D_{in}$.
2. Mean distance from the observation to all other observations in the nearest cluster. Let’s denote it by $D_{out}$.

After calculating the above two distances, the silhouette score, **S**, for each sample is calculated using the following formula:

11 $$\begin{aligned} S = \frac{(D_{out} - D_{in})}{max(D_{in},D_{out})} \end{aligned}$$

The silhouette score varies from − 1 to 1, where 1 means clusters are clearly distinguished and -1 means clusters are assigned in the wrong way. The value 0 means the distance between clusters is not significant.

### Zones and clusters

In the tested building, each floor had multiple thermostats which were connected to variable air volume (VAV) terminal units. VAV terminal units are zone-level flow control devices. Thermostats that are connected to one VAV unit control by that VAV unit and can be considered as one zone. Based on the number of VAV units, we could identify different numbers of zones. After the inspection, we recognized that there are 8, 10, and 10 zones on floor 1, floor 2, and floor 4 respectively. We considered those numbers of zones as the numbers of clusters that we want from the cluster analysis, and compared those cluster results with the actual zones. It helped us identify whether those sensors within zones collect similar data or not, and whether having one thermostat for each zone is reasonable or not.

We clustered building floors based on k-means and two different time series clustering algorithms. From each clustering method, three different results were generated using temperature, temperature + relative humidity, and temperature + relative humidity + pressure. Then, the similarity between zone labels and cluster labels are compared using the ARI, NMI, and AMI similarity matrices. These similarity measures are mainly used to do a pairwise comparison of two cluster results.

Then to determine the minimum number of thermostats, we found the optimal number of clusters using three clustering methods with different variables as discussed earlier. Silhouette score was used to evaluate those cluster results and identify the best method to cluster the building environment.

## Results

### Descriptive analysis

**Fig. 2:**
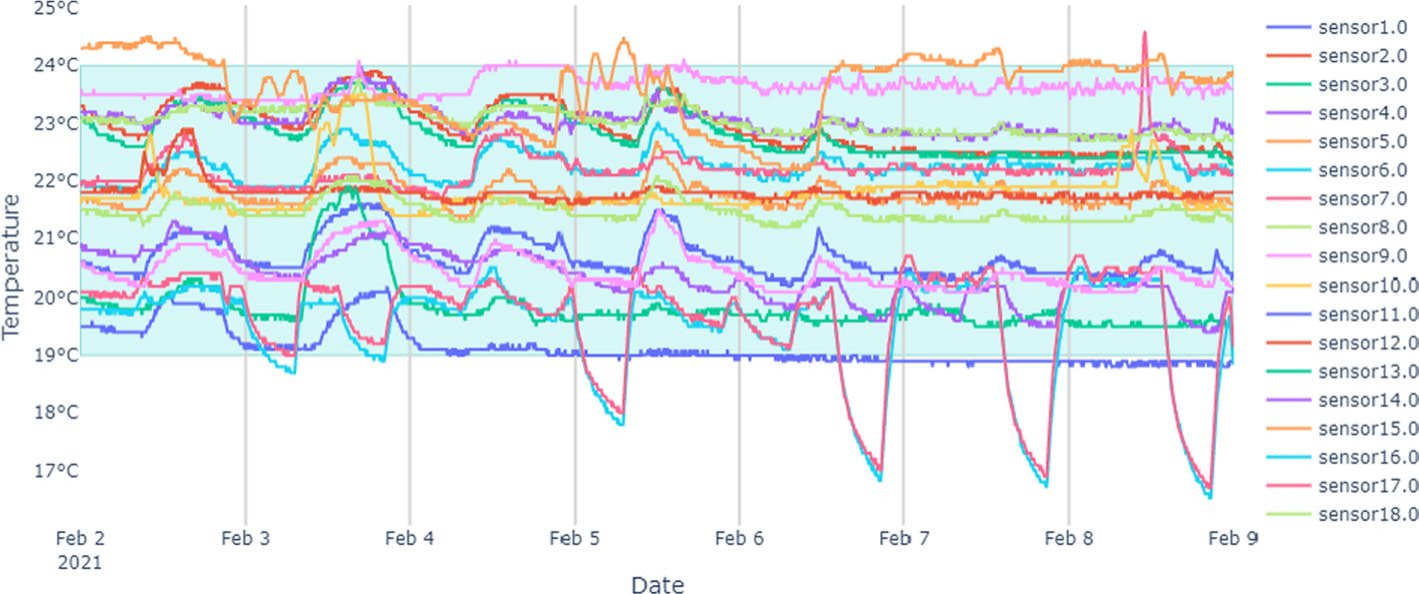
Temperature variations over the entire test period for floor 1. Green color box shows the comfortable temperature range

Before starting the cluster analysis we analyzed the temperature trends of each sensor to identify any anomalies and to understand the building’s current condition. For an example, Fig. 2 shows temperature variations over time on each sensor. Based on ASHRAE (American Society of Heating, Refrigerating and Air-Conditioning Engineers) 55 standards  (ASHRAE 2017), the comfortable temperature range is between 19 and 24$^\circ$C, illustrated by the highlighted region. The majority of temperature values were measured within this comfortable range, except sensors 16 and 17, both of which were located in the same office.

**Fig. 3:**
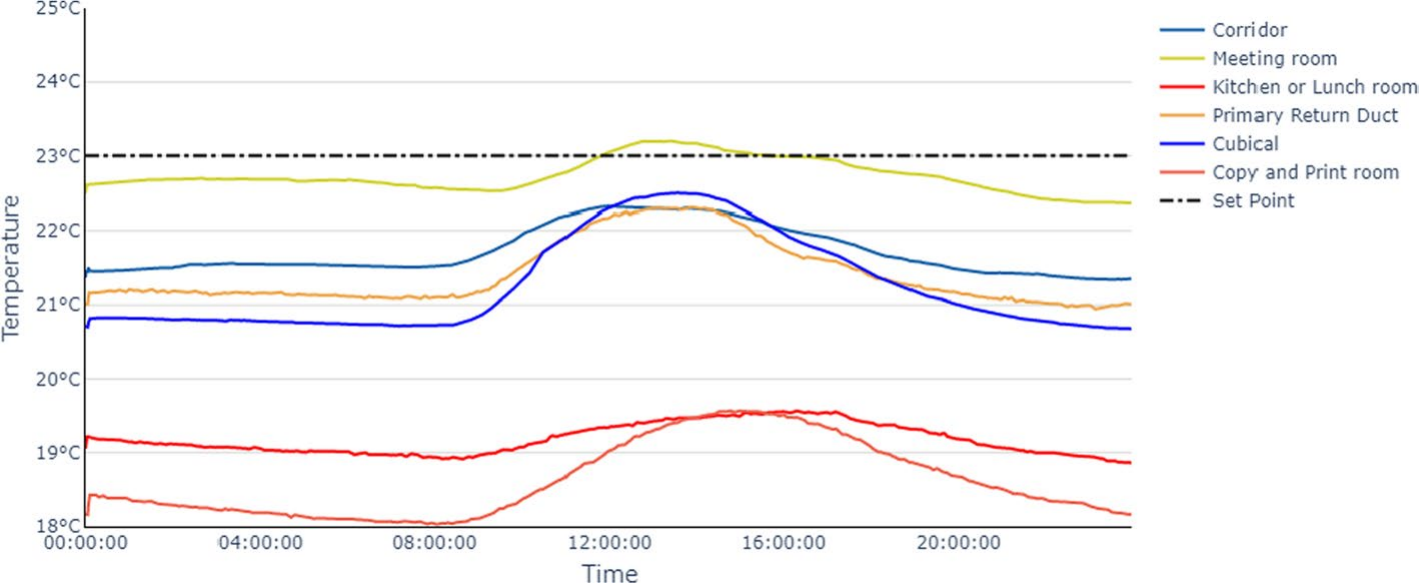
Average temperature over one average day for floor 4. Dash line shows the set point (expected temperature) of this floor

Then we averaged the temperatures on each floor, per location to illustrate the temperature trends over one average day. Figure 3 appears to indicate temperature trends corresponding with occupancy, beginning to increase at 09:00 am and decrease in the afternoon.

**Fig. 4:**
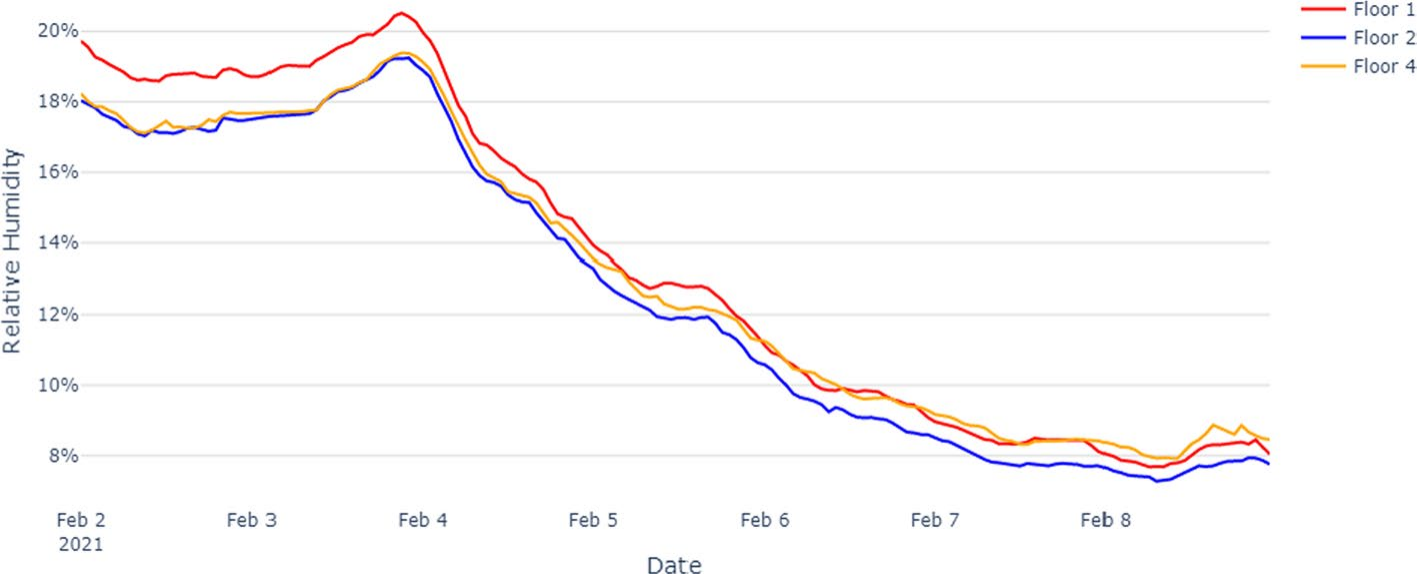
Average relative humidity per floor, over the entire test period. Building shows very low RH values based on ASHRAE 55 standards

Relative humidity (RH) data of all the sensors within each floor showed similar variations with time. Hence we compared floor-wise RH variations during the test period. Figure 4 highlights that floor 1 is typically more humid than floor 2 and floor 4. Based on the ASHRAE 55 standards, a comfortable RH range is between 20 and 60%, but this building showed very low RH values.

**Fig. 5:**
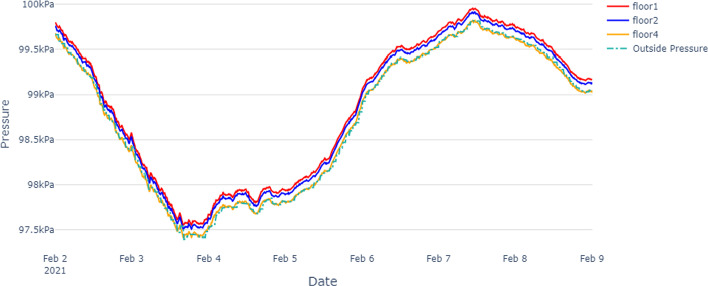
Average pressure per floor, over the entire test period with outside pressure

The average air pressure per floor will depend on many factors, specifically outside weather conditions. When the weather is cold outside, the warm buoyant indoor air tends to rise to the top of the building generating increased pressure in the upper levels, and relatively low pressure in the lower levels. This is known as the stack effect  (Miller 2019; Mijorski and Cammelli 2016). Based on the collected air pressure data of this building, Fig. 5 shows the floor-wise pressure distribution, and it indicates that floor 1 has consistently higher pressure than floor 2 and floor 4. This may represent the reverse stack effect.

### Clustering based on number of zones

**Fig. 6:**
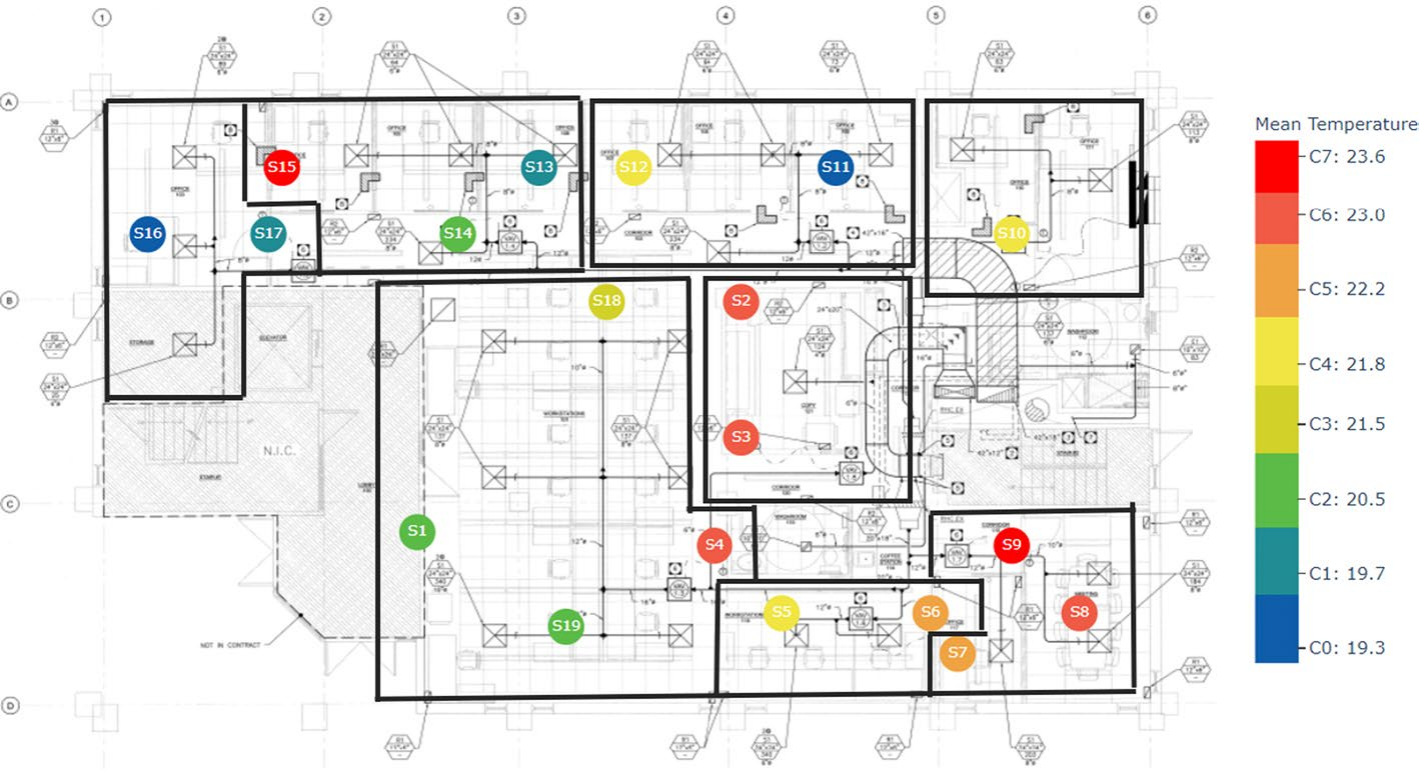
K-means cluster results with zones in floor 1. Black boxes indicate zones while different colors indicate each clusters. Legend shows the mean temperature values of each cluster

**Fig. 7:**
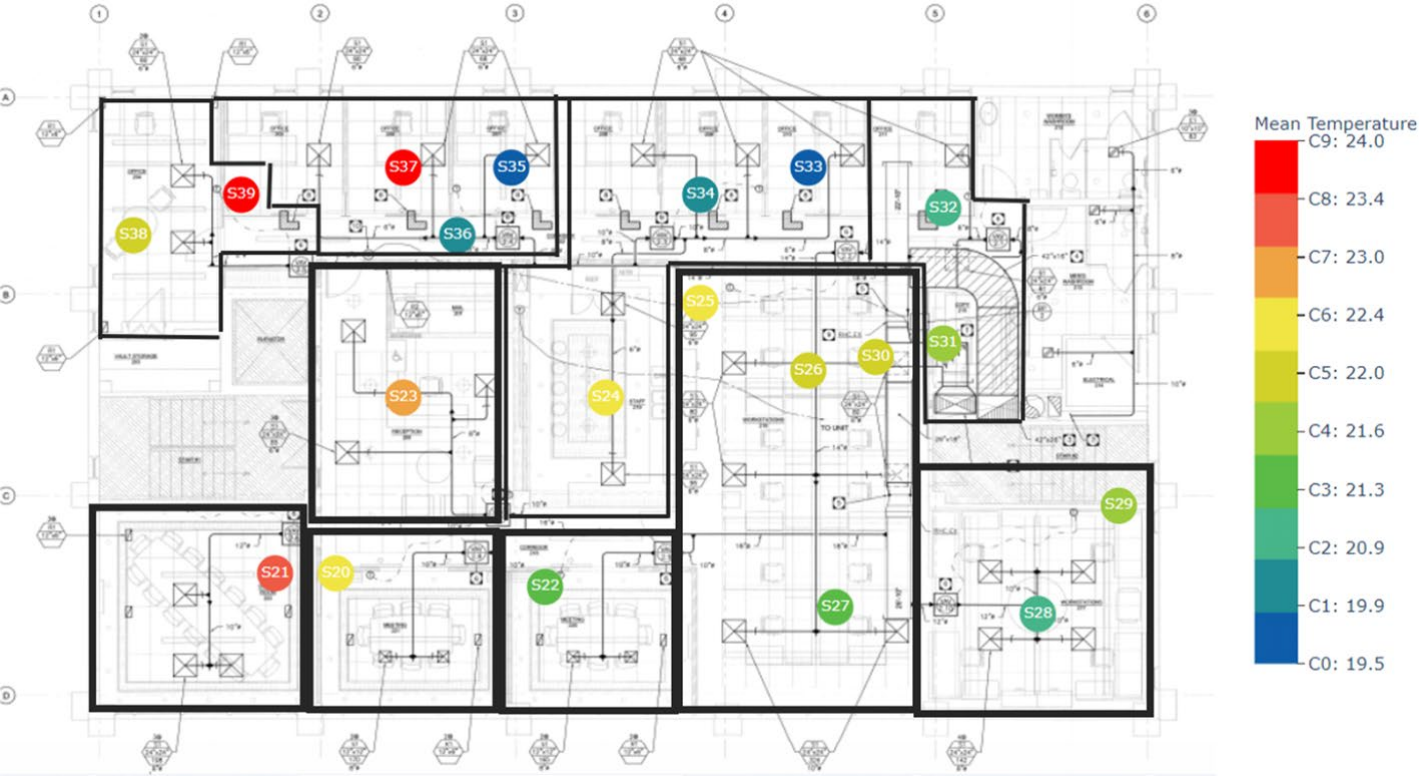
K-means cluster results with zones in floor 2. Legend shows the mean temperature values of each cluster

**Fig. 8:**
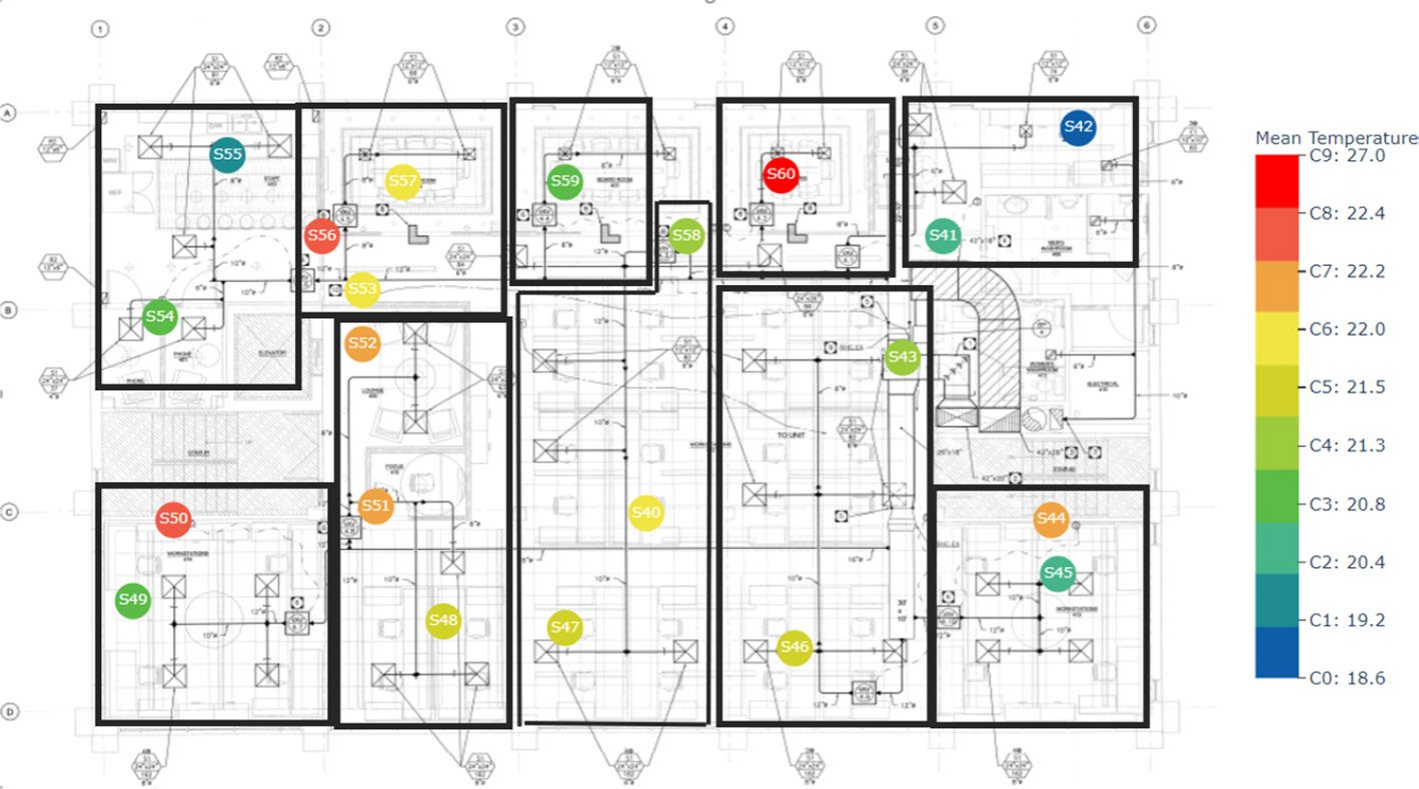
K-means cluster results with zones in floor 4. Legend shows the mean temperature values of each cluster

As discussed in "Material and methods", we clustered each floor using three different methods by considering three different variables. We clustered sensor data from the ground floor, second floor, and fourth floor separately. For example the following Figs. 6, 7, 8 illustrate the k-means clustering results based on the temperature in three different floors. Different zones are indicated by black color borders. The legend of each floor map shows the mean temperature of each cluster from lowest to highest. Blue color nodes indicate the sensors with the lowest mean temperature, while red color nodes indicate the sensors with the highest mean temperature.

Then we calculated similarity matrices to measure the similarity between the zone labels and cluster labels. Table 3 shows the ARI, NMI, and AMI scores for each cluster results which were generated by different clustering algorithms.

**Table 3:** Similarity scores when comparing zoning labels with cluster results

Variables	Floor	K-means clusters			Time-series clusters (Ward, Euclidean)			Time-series clusters (average, correlation)		
		ARI	NMI	AMI	ARI	NMI	AMI	ARI	NMI	AMI
Temperature	Floor1	0.03	0.61	0.05	0.09	0.63	0.12	0.12	0.61	0.2
	Floor2	0.004	0.68	0.01	0.25	0.75	0.28	0.02	0.65	0.03
	Floor4	0.07	0.69	0.09	0.22	0.73	0.25	0.11	0.69	0.18
Temperature + humidity	Floor1	0.07	0.62	0.1	0.18	0.7	0.27	0.24	0.71	0.38
	Floor2	− 0.08	0.64	− 0.1	0.06	0.69	0.08	0.12	0.69	0.25
	Floor4	0.08	0.66	0.1	0.13	0.68	0.15	0.07	0.66	0.11
Temperature + humidity + pressure	Floor1	0.06	0.61	0.09	0.24	0.72	0.31	0.17	0.65	0.26
	Floor2	− 0.09	0.63	− 0.11	0.06	0.69	0.08	0.07	0.68	0.11
	Floor4	0.03	0.66	0.1	0.13	0.68	0.15	0.19	0.72	0.27

### Clustering based on optimal number of clusters

After generating clusters based on the number of zones within each floor, we considered clustering based on the optimal number of clusters. In k-means and time series clustering methods we used elbow plot and silhouette scores to identify the optimal number of clusters, respectively. Table 4 shows the optimal number of clusters and silhouette score for each clustering algorithm.

**Table 4:** Optimal number of clusters and silhouette score for each clustering method

Variables	Floor	K-means clusters		Time-series clusters (Ward, Euclidean)		Time-series clusters (Average, Correlation)	
		Optimal clusters	Silhouette score	Optimal clusters	Silhouette score	Optimal clusters	Silhouette score
Temperature	Floor1	3	0.66	3	0.78	5	0.71
	Floor2	4	0.61	3	0.62	3	0.68
	Floor4	4	0.58	2	0.88	3	0.61
Temperature + humidity	Floor1	4	0.46	2	0.83	2	0.41
	Floor2	3	0.4	3	0.68	4	0.6
	Floor4	4	0.55	2	0.85	2	0.64
Temperature + humidity + pressure	Floor1	6	0.27	2	0.72	2	0.41
	Floor2	4	0.44	2	0.65	2	− 0.05
	Floor4	4	0.36	2	0.85	4	0.54

## Discussion

Based on the results of cluster analysis with zones, it is clear that time-series cluster results have a better agreement with zones than the k-means clusters. When comparing two methods of time series clustering, the results of Ward’s and Euclidean distance method have higher similarity scores than the results of the average and correlation method. However, the overall results show small similarity scores between clustering and zoning, and there can be multiple reasons to have small similarity score values. One reason is that there can be some lurking, unmeasured variables that affect the clustering and the other one is, that zoning has not done properly.

When discussing the results of the optimal number of clusters, it is clear that we can have a smaller number of clusters with sensors that show similar performances. Hence for this building we could easily reduce number of thermostats. Here, we could also see that time-series clustering method clustered sensors better than the k-means clustering, and Ward’s and Euclidean method showed better performance between two time-series clustering methods. Clustering using only the temperature showed better results than combining humidity and pressure to temperature.

For future work, we plan to collect carbon dioxide (CO_2_) in addition to temperature measurements to evaluate the indoor air quality. Air quality tends to decrease as CO_2_ values increase, which has a negative impact on an occupant’s health. Given that humans exhale CO_2_, there exists a connection between occupancy and measured CO_2_ values. Airflow (ventilation) exhausts the breathed air with high CO_2_ concentration, supplying fresh air with low CO_2_ concentration. Temperature and therefore airflow into a space are controlled in part by thermostats, and by evaluating CO_2_ and temperature together we can determine effective clustering based on both temperature (building efficiency) and ventilation (occupant health).

## Data Availability

Data used in this paper is available upon request.
